# Through the Looking Glass: How the COVID-19 Pandemic Changed International Branch Campuses’ Academic Experience and Home Campus Relationship

**DOI:** 10.1177/10283153211070112

**Published:** 2023-02

**Authors:** Rachael H. Merola, Robert J. Coelen, W. H. A. Hofman, Ellen P. W. A. Jansen

**Affiliations:** 1 Faculty of Behavioural and Social Sciences, 3647 University of Groningen, Centre for Internationalisation of Education, Groningen, Netherlands; 2 NHL Stenden University, Centre for Internationalisation of Education, 3647 University of Groningen, Campus Fryslân, Leeuwarden, FR, Netherlands; 3 Research Division Higher Education, Department of Teacher Education, 3647 University of Groningen, Groningen, Netherlands

**Keywords:** qualitative research, transnational education, institutional policy and strategy, student services, teaching, learning and assessment, mobility of programs and people

## Abstract

This study examines how the COVID-19 pandemic has impacted the academic experience at international branch campuses (IBCs) and has changed the relationship between the IBC and the home campus. Semi-structured interviews with 26 leaders, academic staff, and students at seven IBCs in Malaysia revealed that the COVID-19 pandemic has changed the experience at IBCs in unique ways, including collaboration and communication with the home campus; increasing campus-specific resources for student wellbeing; and playing a larger role in student enrollment, recruitment, and mobility initiatives. Findings provide useful insights for higher education institutions (HEIs) engaged in transnational education (TNE).

## Introduction

For decades, international branch campuses (IBCs) have flourished and floundered in all regions of the world—the most recent statistics from the Cross-Border Education Research Team (C-BERT) and Observatory on Borderless Higher Education (OBHE) reveal 305 campuses spanning 77 host countries (C-BERT, 2021; Garrett et al., 2017). Growth has been steady in recent years, with a net increase of 56 IBCs from 2016–2020; 66 from 2011–2015; and 67 from 2006–2010. Most IBCs are in countries in Asia and are founded by institutions based in Western countries (Garrett et al., 2016).

It is difficult to use a “one size fits all” approach to describing IBCs, given the diversity present among these institutions. This study uses the definition put forth by the OBHE and C-BERT in their report on success factors of mature IBCs: “an entity that is owned, at least in part, by a foreign education provider; operated in the name of the foreign education provider; and provides an entire academic program, substantially on site, leading to a degree awarded by the foreign education provider” (Garrett et al., 2017).

An understanding of the student experience at IBCs, which often differs from the experience offered at the home campus (Altbach, 2010), allows universities to develop support services that address the needs of students and attract a diverse student body (Altbach & Knight, 2007). A study by Wilkins (2020) concluded that institutional claims of replicability between the student experience at IBCs and home campuses were “somewhat fanciful”, though overall the experiences may be “largely comparable”, in particular at larger branch campuses. Various studies have found evidence of dissatisfaction with aspects of the academic experience and student support services at IBCs (Ahmad, 2015; Bhuian, 2016; Marginson, 2011; Merola et al., 2021; O’Mahoney, 2014). Given the unique setup and context of IBCs, there is a need to better understand the experience of the students enrolled. In this study, *“academic experience” is defined as “students” interactions with the institution associated with their studies”* (Higher Education Academy, 2014), *including both the informal and formal curriculum* (Leask, 2009).

Nearly all higher education institutions (HEIs) transitioned to online teaching at the onset of the COVID-19 pandemic (Johnson et al., 2020), greatly changing the academic experience for most students. Research on the impact of the COVID-19 pandemic on higher education students shows a variety of effects, particularly related to wellbeing (Debowska et al., 2020; Dodd et al., 2021; Kakuchi, 2021) and the academic experience (Baber, 2020; Basuony et al., 2021; Fatani, 2020; Means & Neisler, 2020). Understanding the effects of the switch to remote learning during the COVID-19 pandemic is important due to the unprecedented nature of the situation: as one study's authors pointed out, “well-planned online learning experiences are meaningfully different from courses offered online in response to a crisis or disaster” (Hodges et al., 2020).

This study focuses on how the COVID-19 pandemic has affected the academic experience IBCs and changed the relationship between the IBC and home campus. No academic research looking specifically at the impact of the COVID-19 pandemic on IBCs has yet been published, to the authors’ knowledge, making this study the first of its kind. It focuses on IBCs located in Malaysia from universities in the United Kingdom (UK) and Australia, for reasons described in the following section.

## The Malaysia Context

Malaysia was selected as the host country in this research because it is often regarded as an “education hub”, due in part to favorable government policies toward TNE as well as a large student market. Malaysia accounts for 40% of enrollments of ASEAN students that study abroad within the region (Atherton et al., 2018), and TNE management processes in Malaysia are “generally well developed”, according to an analysis by the UK's Quality Assurance Agency for Higher Education (QAA, 2020).

Presently, Malaysia hosts twelve international branch campuses, of which five are campuses of institutions in the UK, three are campuses of Australian institutions, and the others are campuses of institutions in China, France, Ireland, and Singapore. The UK and Australia were selected for this study due to these nations’ high levels of TNE activity: the UK alone has 36 IBCs in 18 countries (Garrett et al., 2017). Malaysia's historical ties to the UK has resulted in compatibility between the two education systems, allowing UK universities to offer almost identical programs without making major adaptations to the local context.

Australian universities currently operate twenty IBCs in ten countries, and the nation is widely considered an early pioneer in IBC development, accounting for much of the growth in the 20th century (Garrett et al., 2017). Of the 120,000 students worldwide enrolled in Australian TNE programs in 2019, nearly 20,000 (17%) were enrolled in programs offered in Malaysia (Australian Department of Education, Skills, and Employment, 2019). The geographic proximity and longstanding TNE links to Southeast Asia have attracted some Australian universities to open IBCs in Malaysia.

## Theoretical Foundations

This study is informed by Astin's Theory of Student Involvement (1999), which postulates that students are at the center of their learning experience. For development and growth to occur, students must face challenges in their student life while having access to university and social support. SI theory views the role of the campus as providing students with the opportunity to encounter and engage with new ideas, people, and routines, proposing that each campus should be tailored to the needs of the students enrolled (Pascarella & Terenzini, 2005). This recognizes the importance of understanding the unique academic experience at international branch campuses and examining how it is being impacted by the COVID-19 pandemic.

The Global Integration–Local Responsiveness (I-R) framework analyses the tension between integration and differentiation in multinational subsidiaries (Prahalad & Doz, 1987). Global integration refers to “the degree to which the company is able to use the same products and methods in other countries,” whereas local responsiveness refers to “the degree to which the company must customize their products and methods to meet conditions in other countries” (Lumens, 2021). Within this framework, IBCs are viewed as multinational subsidiaries that compete in local markets while retaining strong ties to the parent company (Brock & Siscovick, 2007).

Previous research using the I-R framework revealed a central challenge of IBC operations to be balancing the need to localize teaching and learning while providing an educational experience equivalent to the one offered at the home campus (Healey, 2018). For example, a joint venture partner may prioritize profit over quality; a host government may make certain coursework mandatory; or the home campus may resist localizing the curriculum. To this end, this research seeks to better understand the relationship between the IBC and the home campus and how it is being shaped by the COVID-19 pandemic.

## Research Questions

How is the COVID-19 pandemic changing the academic experience at IBCs?

How is the COVID-19 pandemic changing the relationship between the IBC and the home campus?

## Methods

### Participants

Twenty-six interviews were conducted virtually with leaders, academic staff, and students at the campuses of seven international branch campuses of UK and Australian universities operating in Malaysia. These individuals were selected using purposive sampling, having been identified by the researchers using their publicly available credentials as possessing relevant insight into the research questions due to their roles and experiences.

While other stakeholders (i.e., alumni, prospective employers, and staff from the home campus) were considered for inclusion, the research seeks the insights from those who are located on the branch campus, with direct experience with the academic operations and perception of the relationship with the home campus. Including leaders, academic staff, and students in the sample was a way to triangulate data and better understand the various perspectives that shed light on the research questions (Cohen et al., 2011). Limitations of the sample are discussed later in the paper.

The sample included six heads or senior leaders of IBCs (Pro-VCs, Presidents, Vice-Presidents, and CEOs), eight academic leaders (Provosts, Deans, and Heads of faculties, departments, or schools), three junior or mid-career academic staff (professors and lecturers), and nine undergraduate students. Table 1 displays the sample characteristics and interview schedule.

**Table 1.** Interviewee Characteristics and Interview Schedule.

Institution	Gender	Position type	Faculty/discipline affiliation	Interview date
1	F	IBC Leader	NA (Leadership role)	10-Feb-21
1	M	Academic Leader	Engineering and Science	20-Apr-21
1	F	Student (Second Year)	Finance	4-Mar-21
1	M	Student (Final Year)	ICT	10-Mar-21
2	M	Academic Leader	Engineering and Science	22-Apr-21
2	M	Academic Staff	Energy	18-Mar-21
2	F	Academic Leader	Psychology	14-Apr-21
2	M	Student (First Year)	Actuarial Science	26-Oct-21
3	M	Academic Leader	Engineering/IT	17-Feb-21
3	F	IBC Leader	NA (Leadership role)	3-Mar-21
3	M	IBC Leader	NA (Leadership role)	1-Mar-21
3	F	Student (Second Year)	Civil Engineering	6-Nov-21
4	M	IBC Leader	NA (Leadership role)	10-Feb-21
4	F	Student (First Year)	Law	2-Mar-21
4	F	Academic Staff	Psychology	24-Feb-21
5	M	Academic Leader	NA (Leadership role)	30-Mar-21
5	M	Academic Leader	Education	15-Apr-21
5	F	Academic Staff	Education	29-Mar-21
5	F	Student (Final Year)	Finance and Accounting	27-Oct-21
5	F	Student (Final Year)	International Business	07-Nov-21
6	M	IBC Leader	NA (Leadership role)	29-Mar-21
6	F	Academic Leader	Design	5-Apr-21
6	F	Student (Final Year)	Biotechnology	3-Nov-21
7	F	IBC Leader	NA (Leadership role)	15-Mar-21
7	M	Academic Leader	Business and Finance	30-Apr-21
7	M	Student (Third Year)	Electrical Engineering	2-Nov-21

The identity of individuals and of the IBCs are anonymized or pseudonymized (for example, the President of an IBC from Australia in Malaysia will be referred to as “the head of an IBC”). Other identifying information is excluded, anonymized, or pseudonymized.

Researchers used semi-structured interviews to explore the research questions to allow for less bias and greater response flexibility (Cohen et al., 2011). The length of the interviews was adjusted according to experience of each interviewee and time constraints. Interviews lasted between 23 min and 65 min.

### Data Collection

The interview instrument contained questions organized by categories and subcategories relevant to the research questions. The categories included *IBC Context and Operations*, *Academic Experience at IBC*, and *Impact of the COVID-19 Pandemic*.

Interviews were conducted remotely via BlueJeans, Zoom, and Microsoft Teams—all of which are secure video conferencing platforms—as the COVID-19 pandemic made in-person interviews impossible. Audio of the interviews was recorded and transcribed. The study was approved by the Ethics Committee of the University of Groningen, and interviewees gave consent prior to participation.

The number of interviews conducted was determined by saturation, which is a frequently used criteria for qualitative rigor referring to the point in data collection at which no additional data are being found that allow the researcher to develop properties of the category (Fusch & Ness, 2015; Guest et al., 2006).

### Data Analysis and Coding Procedure

To efficiently store and organize data, the interview audio and transcripts were uploaded to NVIVO. In addition, researchers wrote memoranda to record any methodological notes or reflections.

To begin the coding process, a small number of provisional codes were developed based on the theoretical foundations, research questions and interview guide (Saldana, 2015). The interviews were then perused iteratively, which allowed the codes to be applied and/or refined. The resulting set of codes was then applied to all interviews. Table 2 contains the codes used to classify information in relation to the two research questions.

**Table 2.** Codes Used to Analyze Interviews.

Topic	Main codes
Context and operations	Changes at IBC since foundation
	Current research at university
	Hiring practices at IBC and at home campuses
	How IBC fits into intl strategy of university
	How IBC success is measured
	Level of autonomy
	Reason for creation
	Role in host country education system
	Role of alumni
	Student and staff mobility btwn campuses
Academic environment	Differences in student support services
	Measure of student engagement
	Student mobility
Academic offering	Collaboration between campuses in course delivery
	Contextualization of content
	Differences between campuses
	How courses are localized
	Perception of academic equivalency
	Process of localizing courses
Learning outcomes	How they are measured and evaluated
	How they are standardized
	Role of employability
Teaching quality	How and to what extent it is monitored
	Role of student feedback
COVID-19 impact	Differential effect on campuses
	Effect on academic offering
	Effect on academic staff
	Effect on comm and collab btwn campuses
	Effect on course delivery
	Effect on HR policies
	Effect on QA and student feedback
	Effect on staff recruitment
	Effect on student enrollment
	Effect on student experience
	Differences in approaches
	Effect on student mobility
	Effect on student recruitment
	Effect on student well being
	Lasting changes from COVID19
	Shifts in role of IBC in intl strategy

After the transcripts had been coded, qualitative analysis to explore each code was conducted. Analysis of each code revealed themes, which were then organized by research questions. For research question 1, the themes that emerged at IBCs were:

- Pre-existing online learning and networks made IBCs well-placed to quickly switch to online delivery
- Student disappointment with halted mobility and lack of in-person academic and social interaction with peers
- Student desire to continue asynchronous online modalities (i.e., recorded lectures available online)
- Expanded services and resources provided by the IBC to support student wellbeing
- Increased effort on the part of the IBC to foster a sense of community

For research question 2, the themes that emerged were:

- More inter-campus and intra-campus collaboration in teaching
- IBCs playing a larger role in enrollment, recruitment, and mobility initiatives of the university
- IBCs helping to minimize the impact of halted/reduced student mobility on the university
- Progression of a university vision of campuses as equal parts of a global university

A content analysis was performed in which the frequencies of themes were counted by the number of interviewees that expressed that sentiment. This gave a sense of which themes were most prevalent among interviewees (Onwuegbuzie et al., 2009).

## Results

Qualitative data analysis of the interviews revealed themes that elucidate the research questions. These are presented below.

### RQ1: How is the COVID-19 Pandemic Changing the Academic Experience at IBCs?

All interviewees agreed that the COVID-19 pandemic, and the resulting switch to online learning, has greatly impacted the academic experience at IBCs. In particular, interviewees expressed a belief that their campus was well placed for a quick switch to online delivery due in large part to the pre-existing online learning, infrastructure used for teaching and learning at the IBC. For example, a professor noted that “we used a learning management system (LMS) even before COVID, so we just went back and used it… the transition wasn't so difficult compared to universities that did not have a platform to fall back on.” The leader of an IBC explained that “normally change at universities is relatively slow, but our model is ‘one university’ and has always had more of a blended model.”

While the top-down view from the leaders’ perspectives suggested a seamless transition to online learning, academic lecturers and students observed a drop, at least initially, in engagement and teaching effectiveness. One student felt that the quality of the lectures dropped because lecturers could not see the reactions of students who had their cameras off and so could not teach as effectively; several others attributed the initial dip in the effectiveness of the learning environment to what one undergraduate described as “shell-shock”, since the COVID-19 pandemic began abruptly. “I give them props for attempting to deliver the same quality of education as [they did] in person. Granted, it has not been the easiest,” said one student. All students felt that the IBC had effectively solicited and acted on feedback to improve, including, for example, shifting the times at which courses were scheduled in order to allow for breaks between online sessions; offering leniency for IT-related difficulties in completing online assignments or exams; and making lecturers more available to students to answer questions outside of class time.

Content analysis points to a differential effect of the COVID-19 pandemic on campuses of the same institution, often resulting from different national regulations. For example, Malaysia retained the requirement that counseling training be conducted in person, leading to delays in completing qualifications for some students at the Malaysia campus that were not experienced by students at the UK or Australia campuses. One student who was on an exchange program at the Australia campus at the start of the pandemic felt that the movement control order was stricter in Malaysia, noting that “the Malaysia campus was totally closed, whereas in Australia, when the lockdown wasn't in place students could still go to campus for labs.”

On the other hand, two of the leaders of IBCs specifically mentioned that they felt the Malaysia campus was a better place to experience the pandemic than other campuses. One leader declared, “Malaysia adapted quickly. We just got on with it, and did some really great work, whereas in the UK, they took a bit longer to adapt.” The onset of the pandemic prompted one student to transfer from a large research university in Australia to an IBC in Malaysia—his home country. In addition to being closer to family and cheaper, he felt that the IBC had superior online infrastructure to the Australian university where he was studying pre-COVID.

Notably, all students were positive about the increased availability of asynchronous online learning modalities that resulted from the pandemic. Students valued being able to access lectures at any time and hoped this feature would remain. However, as one student pointed out, “it takes a lot of self-discipline to actually re-watch lectures” and, in his opinion, in-person classes prevent students from procrastinating and eventually becoming overwhelmed with work. Students were pleased about the increased online interaction with students on other campuses—for example, the ability to post questions on a Blackboard forum shared by students enrolled in the course across all campuses. This helped foster the feeling that the academic experience is the same across all campuses—or, as one student put it, “if you are suffering, you know the other guy in Australia is also suffering.”

Despite understanding the reasons for it, students expressed disappointment with the lack of in-person learning and the inability to pursue mobility plans. Four students had been forced to cancel plans to study at another campus of the university due to the COVID-19 pandemic; only one had been able to carry out his plan to spend a year at the UK campus (the other four students had not had plans to study at another campus). One of the Malaysian students had enrolled in the IBC in 2020 instead of the home campus due to her parents’ desire for her to stay close to home during the pandemic. While she felt “going abroad is a waste of money right now,” she admitted “honestly, I am waiting for things to change. Just being at home, studying, doesn't give much of a university experience.” For the five Malaysian students interviewed, enrolling at an IBC in their home country is a way to have an international education experience.

Relatedly, the COVID-19 pandemic has highlighted the importance of fostering a sense of community, both online and in person, that is unique to the IBC. “The lack of human interaction is really serious because we are staying at home, and university is the place where you build your network and meet new people, and we haven't gotten than experience like pre-COVID students did,” explained one student. A student from Pakistan explained that for students from outside Malaysia, “our homesickness goes away when we meet our friends, so it was very difficult to talk to walls and not meet friends during the pandemic. That played a role for some in not performing well academically.”

Interviews revealed all of the IBCs made efforts to maintain a sense of community online during the COVID-19 pandemic—for example, creating a weekly bulletin with photos from campus, keeping students up to date using social media, and holding events like graduation ceremonies online; however, leaders, staff, and students alike acknowledge that it is difficult to replace the benefits of being together physically. One head of an IBC remarked that “even with all this great tech, we cannot replace that sense of community… there are benefits to being physically together on campus, doing activities together, being part of a community.” Likewise, all students felt that there was no effective substitute for in-person interaction with peers.

The COVID-19 pandemic has prompted IBCs to develop more campus resources for student mental health and academic support, as opposed to relying on those of the home campus. IBCs dedicated more resources to support students, including, for example, hiring mental health counselors, launching wellbeing seminars, opening a mental health hotline, and appointing a virtual wellbeing officer. Students were supported in their daily lives as well—for example, a student from Saudi Arabia recounted that the university helped students living on campus by providing free food vouchers and food delivery for one month, despite it being summer break. Overall, the pandemic prompted IBCs to focus more energy and resources on mental health and wellbeing, rather than relying on the home campus or simply not having them available.

Interviewees also reported initiatives specific to the IBC to offer more academic support to students. A lecturer reported that the IBC now required weekly virtual check-ins between students and academic tutors. The head of Engineering at an IBC created a temporary “academic safety net” to allow students to erase any failing grades during the COVID-19 pandemic. Several academic staff divulged offering more lenient grading; one noting that it was “the least that could be done” given all that students were going through. Students who were asked did not feel that the grading had become more lenient, however. An engineering student pointed to the opposite, saying “There are a lot of changes in how the questions are being asked in exams” and that the online versions are harder than the physical versions.

### RQ2: How has the Relationship between the IBC and the Home Campus changed due to the COVID-19 Pandemic?

All leaders and academic staff interviewed reported that the COVID-19 pandemic had resulted in increased inter-campus and intra-campus collaboration in teaching. All reported having increased meetings with counterparts at the home campus. Additionally, in many cases, universities used IBCs to continue operations during the COVID-19 pandemic, leveraging resources to respond and adapt to the crisis. For example, one interviewee recounted that the home campus had to look to the Malaysia campus to help deliver classes to students; another shared that academic staff at all campuses had pooled together to mark exam papers.

The pandemic's disruption to established teaching routines made academic staff think of new ways to teach effectively, resulting in increased collaboration within and between campuses. A student recounted that lecturers for her engineering course carried out experiments over Zoom for them to observe, since the students themselves could not access the labs on campus. A lecturer at an IBC pointed out that, prior to the pandemic, “we were kind of doing our own thing, and if we were to talk it would be about other things. The move to online has made us talk more about teaching, and there is a lot more collaboration in terms of teaching.” Because IBCs and home campuses moved online at the same time, academic staff across campuses collaborated to find ways to create an engaging learning experience.

Many interviewees stated that the COVID-19 pandemic has highlighted the potential of IBCs to offer pathways to, and through, the university. In particular, interviewees noted plans for IBCs to play a larger role in enrollment, recruitment, and mobility initiatives, including 2 + 1 programs, foundation and pathway programs, and programs with built-in mobility schemes. One IBC head commented that their campus is “at the center of envisioned mobility schemes,” noting that “Since COVID-19, we’ve started to promote that students who are worried about traveling to the UK can spend a year or two on our campus before transferring. We’re giving them more options for their education.” All Malaysian students who were interviewed cited the lower fees and lower cost of living as reasons why they chose to study at an IBC in their home country—a trend that may grow in the wake of price sensitive prospective students and parents.

IBCs appear to have helped minimize the impact of halted/reduced student mobility for the institution. Several academic staff and leaders, when asked, reported that they had absorbed some of the students who were initially planning to study at the home campus, and that this may be an increasingly popular pathway in the future. The head of an IBC hypothesized that some students choose to enroll there rather than at the home campus, with the rationale that if they would have to study online regardless, they would prefer to do so at the IBC, where tuition is cheaper.

For the IBCs in this study, the COVID-19 pandemic has aided the development of a university vision of campuses as equal parts of a global university*.* All interviews with leaders revealed examples of the IBC playing a part in the university's response to the COVID-19 pandemic. These interactions advanced the view of IBCs as strategic footprints that can make valuable contributions to the university. One IBC leader postulated that, perhaps due to the COVID-19 pandemic, the university was becoming more focused on “What is happening in this part of the world, what's changing, and how can we be a part of that. I’m not sure we’ve always been so visionary. In the past the thinking was: ‘a trend is happening; how can we be a part of that?’”

Several interviewees pointed out that the switch to online learning may have had the ancillary effect of “leveling the playing field” between IBCs and home campuses, in terms of the academic experience. One IBC head noted that the move to online learning “helped standardize the academic experience, ensuring that campuses are operating as an integrated institution.” Another interviewee mused that “Before [the pandemic], faculty on the home campus viewed our existence with indifference.” Another said “Now, lightbulbs are going off in Australia, realizing we have a big resource in Malaysia. The campus is not seen as an outpost, but as a group of [a number of] collaborators that can help respond to this crisis.” One university responded to the COVID-19 pandemic by launching an online student community with virtual events open to students from all campuses, which “gave a sense of coherence to the student experiences across campuses,” according to one student.

A recurring theme was that the COVID-19 pandemic has helped shift from an academic model based on duplication to one based on collaboration. “[The COVID-19 pandemic] has brought up how we can use our resources in the most efficient way,” said one IBC head. “It's moved us from the traditional IBC model of ‘*how can we duplicate?*’ to what I am working toward, which is a collaborative model, in other words, ‘*how can we teach the best Bachelor of Science in a global classroom*?’” Another IBC leader explained, “It simply doesn't make sense to have me teaching microeconomics in Malaysia online and you teaching microeconomics in Australia online. Why don't you teach weeks one to seven and I teach weeks eight to thirteen to a combined class? Then we start looking at what I call a global classroom.”

## Discussion

Results speak to the profound impact of the COVID-19 pandemic on both the academic experience at IBCs and the relationship between the IBC and the home campus.

It is notable that interviewees, including students, believed that their institution did a good job at moving online. This is supported by recent studies indicating that university students generally feel their institution was successful in the switch to online and blended learning: for example, a study by the Irish Higher Education Authority found that more than 80% of students in the country felt supported academically during the COVID-19 pandemic (Donnelly, 2021). However, given that some aspects of the move to online learning are likely to be permanent, it is critical that IBCs invest in training academic staff and putting supports in place to successfully offer online and blended learning. The finding that, in some cases, academic staff were being more lenient in marking/grading, as well as the policy of one faculty to erase failing grades, is likely a departure from the academic quality the university seeks to offer and the assurance of academic equivalency across campuses.

Results suggest that fostering a strong sense of community that is specific to the IBC gives students reasons to enroll at the IBC, rather than, for example, enrolling in the degree program online. Interviews with students revealed that they primarily identify as part of their campus community, and secondarily as a part of the wider university community. It stands to reason that IBCs that are not able to maintain a sense of community unique to their campus may struggle to attract and retain students. This is supported by Astin's Student Involvement Theory (1999) as well as research indicating that students’ level of integration is predictive of their satisfaction with their experience (Merola et al., 2019). IBCs that are teaching online must community as a part of the value proposition for students to make enrolling an appealing option.

Results highlighted the potential of IBCs to increase education pathways and mobility options. IBCs aided universities in student enrollment during the pandemic by allowing some students to study at their campus instead of at the home campus, for those that could not do so for various reasons. Universities had begun to promote the various education pathways and programs available more heavily in connection with the IBC, for example, the option to begin a degree at the IBC and then transfer to the home campus. Going forward, universities may leverage the greater quality and availability of online provision to make education pathways and mobility options within the reach of more students.

An indirect effect of the move to online learning may be increased higher education internationalization, defined as “the intentional process of integrating an international, intercultural or global dimension into the purpose, functions and delivery of post-secondary education, in order to enhance the quality of education and research for all students and staff, and to make a meaningful contribution to society” (De Wit & Leask, 2015). HE internationalization has evolved from being conceptualized as a *response* to globalization to an intentional *process* that considers impact on local and global communities (Leask & de Gayardon, 2021). As interviewees noted, the sudden move to online teaching caused academic staff to work together with the intention of delivering high quality education to their diverse student bodies on multiple continents. Virtual academic exchange between campuses, informal online learning between students at different campuses, online social events open to students across campuses, and exchange of best practices in teaching between faculty on different campuses are all examples from the interviews of how the universities are integrating international, intercultural, or global dimensions into education due to the COVID-19 pandemic.

## Conclusions and Limitations

This study offers several takeaways for IBC leaders and academic staff. First, IBCs must find ways to foster a sense of community and belonging that is specific to their campus, and provide support and resources tailored to the needs of their students. Interviewees made clear that an effective replacement for in-person interaction has yet to be found; this is a finding for leaders to take into considering when deciding which aspects of the student experience to offer virtually vs. in person. Additionally, results demonstrate the potential for IBCs to lend versatility to the university in both where and how education is delivered. As necessity is the mother of invention, the COVID-19 pandemic forced IBCs to find new ways to offer education, and as universities reassess their international strategy, IBCs may leverage their location and desirability to play a larger role in the university's enrollment, recruitment, and mobility initiatives. The increased communication between the IBC and home campus can be used to develop more pathways to—and through—IBCs.

Despite measures to improve the trustworthiness of the data, including cross-checking codes and interviews, there are limitations to this research. None of the researchers were able to travel on site to the IBCs included in the study to gather data in person, though they had visited some of the campuses in the past. Data was collected over a nine-month period (February through November 2021), during which time the effects of the COVID-19 pandemic evolved. The academic experience and role of IBCs will continue to develop over time, opening potential for follow up studies.

The decision to focus on one host country (Malaysia) and two home countries (UK and Australia) was deliberate, to avoid the complexities of multiple national regulatory frameworks. However, given the wide geographic presence of IBCs, it would be elucidating to see a greater variety of home and host countries represented. Including a greater breadth of home and host countries would make findings more easily transferable to other contexts. Another angle of inquiry involves comparing IBCs to the home campus, to demonstrate how the pandemic affected these two types of campuses differently. While this study considered how IBCs were uniquely affected by the COVID-19 pandemic, a comparative methodology would highlight differences between the IBC and home campus.

There is inevitable bias in both the participants and the researchers. This research includes some powerful individuals as subjects, who “are well able to deal with the interviewers, to answer and avoid particular questions to suit their own ends, and to present their own role in events in a favorable light” (Walford, 2013). As a result, the views expressed by the IBC leaders may have been carefully calibrated rather than completely candid. The researchers were subject an uneven power dynamic between the researcher and subject; awareness of the existence of interpersonal dynamics and potential bias is one strategy to counter their effects.

The COVID-19 pandemic has drastically changed how higher education is carried out around the world, and stress-tested universities. It has made clear that the landscape of higher education is constantly changing, and institutions must change along with it. In the words of author HG Wells: “adapt or perish, now as ever, is nature's inexorable imperative.” This research suggests that international branch campuses have been able to adapt the academic experience and help the home campus navigate the challenges of the pandemic. As a result, the IBCs in this study have become a more important component of the university's internationalization strategy.
